# Dissecting the Metabolic Costs of Uphill and Downhill Walking

**DOI:** 10.1111/sms.70389

**Published:** 2026-10-26

**Authors:** Luke N. Jessup, Luke A. Kelly, Andrew G. Cresswell, Glen A. Lichtwark

**Affiliations:** ^1^ School of Human Movement and Nutrition Sciences University of Queensland Brisbane Queensland Australia; ^2^ School of Biological Sciences University of California Irvine Irvine California USA; ^3^ School of Health Sciences and Social Work Griffith University Gold Coast Queensland Australia; ^4^ Australian Centre for Precision Health and Technology Griffith University Gold Coast Queensland Australia; ^5^ School of Exercise and Nutrition Sciences Queensland University of Technology Brisbane Queensland Australia

**Keywords:** biomechanics, energetics, locomotion, muscle, work

## Abstract

Work‐ and collision‐based models of locomotion relate the body's mechanical work requirements to metabolic energy expenditure at a system level, but do not capture the underlying force and work demands of muscle that drive energy use. Energy is liberated as heat to activate muscle and maintain force production (“force‐related costs”) and as heat and work to shorten or lengthen active muscle (“work‐related costs”). We collected experimental inverse dynamics, electromyography, and ultrasound data from the main power‐producing lower‐limb muscles during walking at slopes spanning ±15%. These data were used to evaluate a musculoskeletal modeling approach that simulates muscle states, inputs them to a phenomenological energy cost model to estimate metabolic power, and partitions force‐ and work‐related costs. Simulated metabolic power was compared with indirect calorimetry. Summing simulated metabolic power across only the eight muscles evaluated experimentally correlated more strongly with experimental metabolic power than summing across all modeled muscles (adjusted *R* ^2^ = 0.95 vs. 0.86), so analyses focused on this subset. Both approaches differed from experimental metabolic power in absolute terms, so results are interpreted as relative changes across slopes. Simulated force‐related costs increased at steeper inclines and declines and were moderately correlated with mean joint moments (adjusted *R* ^2^ = 0.67), with unexplained variance likely reflecting muscle operating lengths. Simulated work‐related costs increased as slope became more positive and were strongly correlated with net joint work (adjusted *R* ^2^ = 0.95). Knee joint mechanics seem particularly indicative of these costs. Incorporating a force‐related cost term may offer a more explanatory link to muscle mechanical demands than work‐ and collision‐based models alone.

## 1 Introduction

Locomotion is rarely performed at steady state on level ground; instead, mechanical energy (work) requirements tend to fluctuate. While we can anticipate changes in these work requirements, the precise mechanical and energetic consequences remain poorly understood. This information is needed to form a cohesive biomechanical understanding of how energy is added to or absorbed by the musculoskeletal system when we accelerate or brake, or locomote uphill or downhill, on surfaces of different compliance, Aor with added or reduced mass. This study focuses on sloped locomotion, where changes in surface gradient proportionally alter mechanical work requirements, making it an ideal model for examining the relationship between these requirements and metabolic cost.

Biomechanical models of energy expenditure during sloped locomotion typically quantify positive and negative work and assume mechanical efficiencies consistent with shortening and lengthening muscle contractions, respectively [1, 2, 3, 4]. Likewise, collisional losses of energy between the absorptive and generative phases of stance have been used to estimate the energy that muscular work must replace [5, 6]. Minetti et al. [7] and Dewolf and Willems [5] used work‐ and collision‐based models to explain whole‐body (“system‐level”) changes in the cost of transport (metabolic cost per distance traveled) across surface gradients in walking and running, respectively. The cost of transport of uphill walking increases with slope, becoming a linear relationship at slopes exceeding +15%; whilst for downhill walking, there is initially a reduction in cost of transport, followed by an increase at slopes steeper than −10%, with a shallower linear trend beyond −15% [2, 4]. The linear regions correspond to the mechanical efficiencies of pure shortening and lengthening contractions. The minimum corresponds to the point where mechanical energy exchange is greatest and energy generated equals energy absorbed during collision [5, 8]. The shallower, nonlinear regions may reflect spring‐ and pendular‐like energy‐saving mechanisms [4, 9] However, these frameworks do not describe the mechanical demands on muscle that directly determine energy expenditure. Their assumptions about muscle efficiency, and about energy transferred between body segments or stored and returned elastically by muscle and tendon, must be adjusted if they are to accurately predict muscle behavior and absolute metabolic cost across conditions and individuals.

Muscle expends energy to generate mechanical work (*w*_CE_), but inefficiencies in energy conversion produce additional heat (*h*_CE_) [10], which depends on the mechanical conditions experienced and therefore influences overall metabolic demand. The main mechanical determinants of muscle energy use are reasonably well understood [11]. During isometric contractions, *h*_CE_ is produced due to ion transport to activate muscle (*h*_A_; “activation” heat) and cross‐bridge cycling to maintain force production (*h*_M_; “maintenance” heat) [12, 13, 14]. These heats scale based on activation level, which is proportional to the force requirements of muscle. *h*_CE_ is also produced when muscle shortens and lengthens whilst active (*h*_SL_; “shortening/lengthening” heat) [10, 15, 16, 17]. At a given activation level, the rate of *h*_CE_ increases with shortening velocity, relative to that of an isometric contraction (where *h*_SL_ is absent). The rate of *h*_CE_ production during lengthening contractions is more complicated, initially decreasing with increased lengthening velocity, but then increasing in proportion to the work done on the muscle to stretch it [18]. *w*_CE_, the integral of force multiplied by the change in length of muscle (positive during shortening and negative during lengthening) [19], combines with *h*_SL_ to represent the work‐related costs of muscle.

Inferring muscle‐level force and work demands is challenging during locomotion, largely due to the elasticity in muscle‐tendon units. Lichtwark and Wilson [9] found that medial gastrocnemius (MG) electromyography (EMG) increased with incline during walking at slopes between ±10% but with no significant change in MG fascicle shortening or shortening velocity due to the substantial elastic contributions of the Achilles tendon. However, based on trends visible in their data (see Figure 3A from Lichtwark and Wilson [9]), together with evidence that the slope–cost relationship becomes increasingly linear beyond ±10% slope, fascicle length changes may be more apparent at steeper slopes.

Few studies have assessed muscle force‐ or work‐related demands during up‐ or down‐hill locomotion. Relative muscle activation is a useful proxy for *h*_A_ and *h*_M_ as it indicates the number of fibers required to be activated to generate force. Lay et al. [20] and Franz and Kram [21] showed that during walking at slopes between ±15%, the mean EMG of MG, soleus (SOL), vastus medialis (VM), rectus femoris (RF), biceps femoris (BF), and gluteus maximus (GMAX) increases with incline, and that of VM and RF also increases with decline. These changes in muscle activity were accompanied by corresponding changes in joint moments. During downhill walking, muscles must absorb mechanical energy and control the body's descent through lengthening contractions, which requires large braking forces and therefore substantial activation, even though little positive work may be performed. Thus, muscle force‐related costs may increase at steeper walking slopes, both uphill and downhill, owing to greater propulsive and braking force demands, respectively. Meanwhile, others have measured joint work during uphill and downhill locomotion, as an indication of whether muscles generate or dissipate net mechanical energy (*w*_CE_). These studies show that the net mechanical energy injected by the ankle, knee, and hip all increase with incline [22, 23, 24]. Thus, muscle work‐related costs are likely to increase as slope becomes more positive, considering also the additional heat produced during active muscle shortening.

Computational musculoskeletal modeling [25, 26, 27] offers a means to explore how movement influences muscle states and their associated energy costs [28]. Koelewijn et al. [29] reported strong correlations between costs modeled from simulated muscle states versus those calculated from indirect calorimetry during sloped walking. They compared seven cost models using a two‐dimensional sagittal‐plane model with eight muscles per leg, at three slopes (−8%, 0%, +8%) and two speeds (0.8 and 1.3 m·s^−1^). Notably, these slopes do not reach the linear regions of the slope‐cost relationship, nor do they encompass the ~−10% cost minimum. Moreover, simulated muscle forces were checked only against literature EMG for level walking and, for slope trends, against another model's predictions (no EMG or fascicle data were collected across slopes). One of the cost models used, originally developed by Umberger et al. [28] and later refined [30, 31], has been validated against direct measures of energy consumption from isolated muscle. It derives *ḣ*_A_, *ḣ*_M_, *ḣ*_SL_, and *ẇ*_CE_ from muscle states to compute the total rate of energy expenditure. These individual cost components can also be analyzed separately to determine their respective contributions. However, the accuracy of this modeling hinges on the fidelity of the simulated muscle states, and the few existing model validation studies show varying levels of agreement with experimental data [27, 32, 33, 34].

In this study, we collected experimental data during walking on slopes between ±15% to evaluate an inverse musculoskeletal modeling approach involving the Umberger cost model. Unlike Koelewijn et al. [29], who compared multiple cost models using a 2D musculoskeletal model over a narrower slope range, we used a 3D model, evaluated simulated muscle states against concurrently measured EMG and ultrasound data, and used a single cost model to partition total cost into its force‐ and work‐related components. We aimed to determine how muscle force‐related (*ḣ*_A_ + *ḣ*_M_) and muscle work‐related (*ẇ*_CE_ + *ḣ*_SL_) costs contribute to total metabolic rate as humans walk steeper uphill and downhill. Note, muscle “force‐related” costs refer to those tied to activating muscle to achieve force requirements, consistent with the terminology of previous literature exploring biomechanical determinants of the energy cost of locomotion [35, 36, 37, 38, 39]. Therefore, the force‐related costs are linked to muscle dynamics—for example, when force potential drops there is a greater requirement for activation to meet the force demands. We hypothesized that muscle force‐related costs would increase on both steeper uphill and downhill slopes, and that muscle work‐related costs would rise as slope became more positive. Thus, we expected that changes in force‐related costs would dominate changes in total metabolic rate during downhill walking, with work‐related costs to play a greater role during uphill walking. We also anticipated that most of the variance in force‐ and work‐related costs would be explained by joint moments and joint work, respectively, with knee mechanics being especially predictive.

## 2 Materials and Methods

### 2.1 Participants

Thirteen healthy participants (5 female, 8 male), aged 30 ± 7 (mean ± SD) years, height 179 ± 9 cm, and mass 76 ± 10 kg consented to participate in this study. This study was approved by, and conducted in accordance with the University of Queensland, Faculty of Health and Behavioral Sciences Low and Negligible Risk Ethics Sub‐Committee (2022/HE000001). This study conformed to the standards set by the Declaration of Helsinki.

### 2.2 Experimental Protocol

Participants walked on a force‐instrumented tandem‐belt treadmill (Tandem Treadmill; AMTI, MA, USA). Treadmill speed was set to each participant's preferred walking speed (1.2 ± 0.1 m·s^−1^), determined during a 3‐min familiarization period in which treadmill speed was varied between 0.8 and 1.5 m·s^−1^ on level (0%) slope. Seven different fixed slope conditions—three downhill (−5%, −10%, −15%), one level, and three uphill (+5%, +10%, +15%)—were performed in a random order during a single testing session. Each trial lasted 5 min and was followed by at least 5 min of rest to ensure full recovery before the next trial. Metabolic energy consumption was recorded continuously throughout each trial, while kinematics, kinetics, and muscle mechanics were simultaneously recorded for 10 s at the 3‐min mark under the assumption that steady‐state walking had been achieved by that time.

### 2.3 Experimental Data

#### 2.3.1 Kinetics and Kinematics

Ground reaction forces (GRFs) were recorded at 2000 Hz from the force‐instrumented treadmill. GRF data were time‐synchronized with 200 Hz motion capture (Vantage, Vicon, Oxford, UK) of the position of 43 reflective markers placed bilaterally on anatomical landmarks on the feet, shanks, thighs, pelvis, and torso [40]. GRF and marker data were filtered using a zero‐lag, fourth‐order low‐pass Butterworth filter with a 10 Hz cutoff, then processed in OpenSim [26]. Using standard OpenSim tools, a model developed by Lai et al. [41] was scaled and used with the marker and GRF data to compute joint kinematics and kinetics. A custom MATLAB (MathWorks, MA, USA) script was used to differentiate joint angles with respect to time to determine joint velocities, which were then multiplied by joint moments to calculate joint powers. Time‐series data were cropped to one gait cycle (from right‐foot ground contact through to the next right‐foot contact). Stride metrics (duty factor and ground contact time) are detailed in the Results (Table 1). Joint moments were averaged, and joint powers integrated with respect to time, over the gait cycle to quantify the mean force and network requirements of each joint, respectively. All experimental metrics were averaged over three gait cycles per condition per participant.

**TABLE 1:** Mean ± SD gait cycle duration (*t*_cycle_) and duty factor across all prescribed walking conditions (−15%, −10%, −5%, 0%, +5%, +10%, +15%). Treadmill speed was held constant across conditions for each participant (1.2 ± 0.1 m·s^−1^). Data averaged across 12 participants.

	−15%	−10%	−5%	0%	+5%	+10%	+15%
*t*_cycle_ (s)	1.14 ± 0.10	1.14 ± 0.08	1.15 ± 0.07	1.17 ± 0.06	1.20 ± 0.08	1.23 ± 0.10	1.20 ± 0.12
Duty factor	0.64 ± 0.01	0.64 ± 0.01	0.65 ± 0.01	0.65 ± 0.01	0.65 ± 0.01	0.65 ± 0.01	0.66 ± 0.01

#### 2.3.2 Muscle Fascicle Dynamics

Experimental measurements of muscle fascicle dynamics, muscle activation, and metabolic energy consumption were used primarily for model evaluation. Further details describing how these data informed the evaluation are provided in the Supporting Information. Two linear‐array B‐mode ultrasound transducers (LV8‐5 N60‐A2, Telemed, Vilnius, Lithuania), fixed to the right leg, imaged LG, SOL, and VL muscle fascicles, with data recorded in EchoWave II software (Telemed). A frame‐by‐frame external trigger enabled data collection at 160 Hz and synchronization with the GRF and motion capture data. Fascicle lengths were tracked in MATLAB using semi‐automated software [42, 43]. Fascicle lengths were normalized to the respective muscle's mean fascicle length across all trials that the participant performed.

#### 2.3.3 Muscle Activation

Surface EMG (MiniWave, Cometa, MO, USA) was used to measure muscle activation from the right leg for LG, MG, SOL, tibialis anterior (TA), VL, RF, BF, and GMAX, with electrodes (Covidien, MA, USA) placed according to SENIAM guidelines [44]. Raw EMG signals were amplified 1000‐fold, hardware filtered with a 10 Hz high‐pass and 1000 Hz low‐pass filter, and recorded at 2000 Hz in Vicon Nexus (Nexus, Vicon, Oxford, UK). Signals were then processed in MATLAB: DC offsets were removed, a high‐pass filter with 25 Hz cutoff was applied, signals were rectified, and a zero‐lag, fourth‐order low‐pass Butterworth filter with a 10 Hz cutoff was applied to produce an envelope [45]. EMG signals were then normalized to the respective muscle's maximum EMG across all trials that the participant performed.

#### 2.3.4 Metabolic Energy Consumption

Oxygen consumption and carbon dioxide exhalation were recorded using a portable spirometry system (Metamax 3B, Cortex, Leipzig, Germany) under standardized conditions (≥ 2 h post‐prandial and prior to any daily caffeine consumption). Resting values were measured during a 5‐min period of quiet standing prior to walking. Steady‐state oxidative metabolism was defined by a plateau in oxygen consumption and a respiratory exchange ratio (RER) below 1.0. These criteria had to be satisfied throughout the final 2 min of each trial, during which data were averaged and converted to gross metabolic power using standard equations [46]. All participants met these criteria for every slope condition, with maximum RER remaining below 1.0 in all trials (mean ± SD: 0.86 ± 0.05; range: 0.76–0.98).

### 2.4 Neuromuscular Simulations

An open‐source model published by Lai et al. [41] was used as the base model for our analyses. This model included 80 Hill‐type muscle‐tendon units (MTUs) and 19 degrees of freedom (6 at the pelvis, 5 in each leg, and 3 in the upper body). We used OpenSim Moco (v1.2.0) [47] to solve the muscle redundancy problem and determine muscle states to achieve the prescribed body kinematics and kinetics. Moco's inverse problem solver employs direct collocation and minimizes the sum of squared controls as its objective function. Due to the computational demands of Moco, we simulated one gait cycle per trial, per participant. The cycle was selected by overlaying each individual cycle's left and right joint moments on the average across the three experimental cycles and visually identifying the cycle most similar in magnitude and shape. This was intended to ensure the simulated cycle was representative of the participant's steady‐state gait in that condition. Although this approach does not capture stride‐to‐stride variability, such within‐condition variability in EMG, fascicle behavior, and joint mechanics appeared small relative to the changes across slopes, as expected given that data were analyzed from periods of steady‐state walking. To compute rates of energy expenditure, the muscle states from Moco were input to the cost model developed by Umberger et al. [28], with modifications as described in [30, 31], using OpenSim's Umberger2010MuscleMetabolicsProbe.

Model refinement and evaluation procedures, detailed in the Supporting Information, involved an iterative, systematic process comparing our experimental data and simulated muscle activations (Figure S1), fascicle dynamics (Figure S2), and whole‐body metabolic powers (Figure S3). Data from one male participant (age 18 years, height 196 cm, mass 84 kg) were excluded due to modeling complications that could not be resolved. Summing the simulated metabolic power across all muscles in the model resulted in large absolute differences compared to experimentally measured metabolic power (Figure S3). In examining the average contribution of each muscle to total simulated metabolic power (Figure S4), stabilizing muscles at the hip joint appeared to contribute excessively. Instead, summing simulated metabolic power across only the muscles evaluated using EMG and ultrasound (LG, MG, SOL, TA, VL, RF, BF, and GMAX) underestimated experimentally measured metabolic power but correlated more strongly with it than the full‐model sum (evaluated muscles: *β* = 4.11, 95% CI [3.87, 4.36], adjusted *R* ^2^ = 0.95; all muscles: *β* = 2.28, 95% CI [2.05, 2.51], adjusted *R* ^2^ = 0.86; Figure S3). Therefore, we included only those evaluated muscles in our analyses of muscle force‐ and work‐related costs.

With our modeling approach evaluated and musculoskeletal simulations performed on our 12 participants, we used the Umberger cost model to partition the contributions of muscle force‐ (*ḣ*_A_ + *ḣ*_M_) and work‐related (*ẇ*_CE_ + *ḣ*_SL_) costs to simulated total metabolic power across the walking conditions.

### 2.5 Statistics

Linear models (LMs) were used to assess the strength of association between (i) total simulated metabolic power (both summed across all muscles in the model and summed across only those muscles for which we had experimental data) and experimentally measured metabolic power, (ii) muscle force‐related costs and joint moments, and (iii) muscle work‐related costs and joint work. Joint moments and joint work were analyzed both for individual joints (ankle, knee, and hip) and for all three joints combined within the regression models. Participant ID was included as a categorical predictor to account for interparticipant variability. Assumptions of linearity, homoscedasticity, and normality of residuals were assessed by visual inspection of residual plots, with no meaningful violations observed. Model fit was quantified using the adjusted coefficient of determination (adjusted *R* ^2^), and for models with a single predictor of interest, the unstandardised regression coefficient (*β*) and its 95% confidence interval (CI) were also reported. We classified adjusted *R* ^2^ values of ≤ 0.35 as indicating weak correlations, 0.36–0.69 as moderate correlations, 0.70–0.89 as strong correlations, and ≥ 0.90 as very strong correlations [48]. As analyses focused on the magnitude of associations (adjusted *R* ^2^ and *β* with 95% CI) rather than null‐hypothesis significance testing, no correction for multiple comparisons was applied.

### 2.6 Use of AI Methods

ChatGPT (OpenAI) and Claude (Anthropic) were used solely for language editing. The authors reviewed all AI‐assisted edits and take full responsibility for the content of this manuscript.

## 3 Results

### 3.1 Stride Metrics

Despite having only their walking speed constrained, participants used a similar gait cycle duration and duty factor across all conditions (Table 1). As treadmill speed was held constant across conditions for each participant (1.2 ± 0.1 m·s^−1^), cadence and stride length followed directly from gait cycle duration, ranging from 98 to 105 steps·min^−1^ and 1.37–1.48 m, respectively.

### 3.2 Metabolic Energy Expenditure

Total simulated metabolic power summed across LG, MG, SOL, TA, VL, RF, BF, and GMAX correlated very strongly with experimental metabolic power (*β* = 4.11, 95% CI [3.87, 4.36], adjusted *R* ^2^ = 0.95; Figure 1A; Figure S5), although costs were underestimated in absolute terms. We observed a progressive increase in total metabolic power from 0% to + 15% slope, and a U‐shaped relationship between total metabolic power and surface gradient at negative slopes, with a minimum at −10% slope—mirroring the trends observed in the experimental data. Muscle force costs (*ḣ*_A_ + *ḣ*_M_) increased at steeper uphill and downhill slopes (Figure 1B), while muscle work costs (*ẇ*_CE_ + *ḣ*_SL_) increased as slope became more positive (Figure 1C).

**FIGURE 1:**
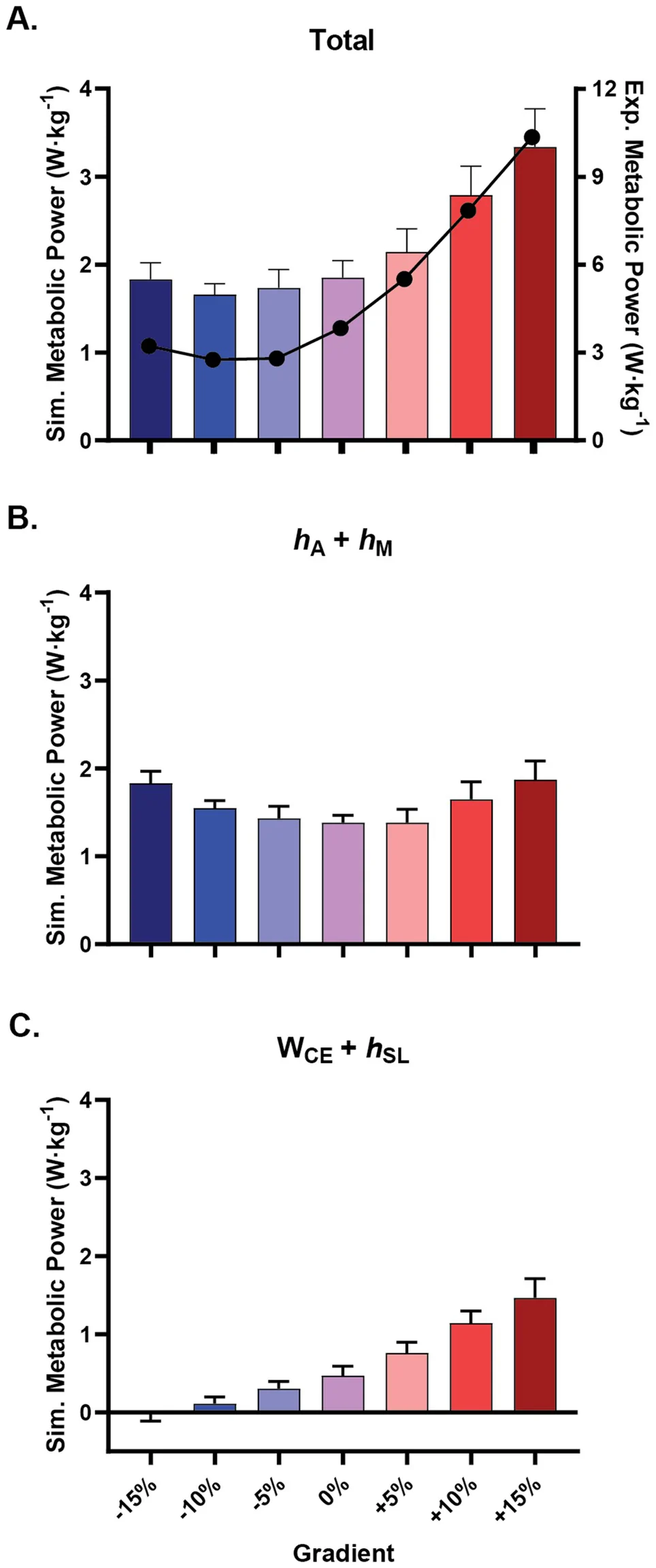
Partitioning mean ± SD simulated net metabolic power (W·kg^−1^) summed bilaterally from lateral gastrocnemius, medial gastrocnemius, soleus, tibialis anterior, vastus lateralis, rectus femoris, biceps femoris, and gluteus maximus into muscle force‐ and work‐related costs across all prescribed walking conditions. (A) Total metabolic power. (B) Force‐related costs (activation heat (*ḣ*_A_) + maintenance heat (*ḣ*_M_)). (C) Work‐related costs (work of contractile element (*ẇ*_CE_) + shortening/lengthening heat (*ḣ*_SL_)). (A) Also includes the mean experimental metabolic power (W·kg^−1^) for each walking condition (circles) on a separate axis. Simulated data are averaged across 12 participants, while experimental data are averaged across seven participants.

### 3.3 Joint Mechanics and Energetics

A U‐shaped relationship existed between mean knee moment and surface gradient, increasing at steeper uphill and downhill slopes (Figure 2), while mean ankle and hip moments showed little change across slopes. Mean knee moment was moderately correlated with muscle force costs (*β* = 1.46, 95% CI [0.95, 1.98], adjusted *R* ^2^ = 0.45), whereas mean ankle (*β* = 0.02, 95% CI [−1.53, 1.56], adjusted *R* ^2^ = 0.12) and hip (*β* = −1.14, 95% CI [−2.13, −0.15], adjusted *R* ^2^ = 0.20) moments were weakly correlated. Including mean ankle, knee, and hip moments as predictors in the regression model explained substantially more variance in muscle force costs than when considered individually (adjusted *R* ^2^ = 0.67) (Figure S6A).

**FIGURE 2:**
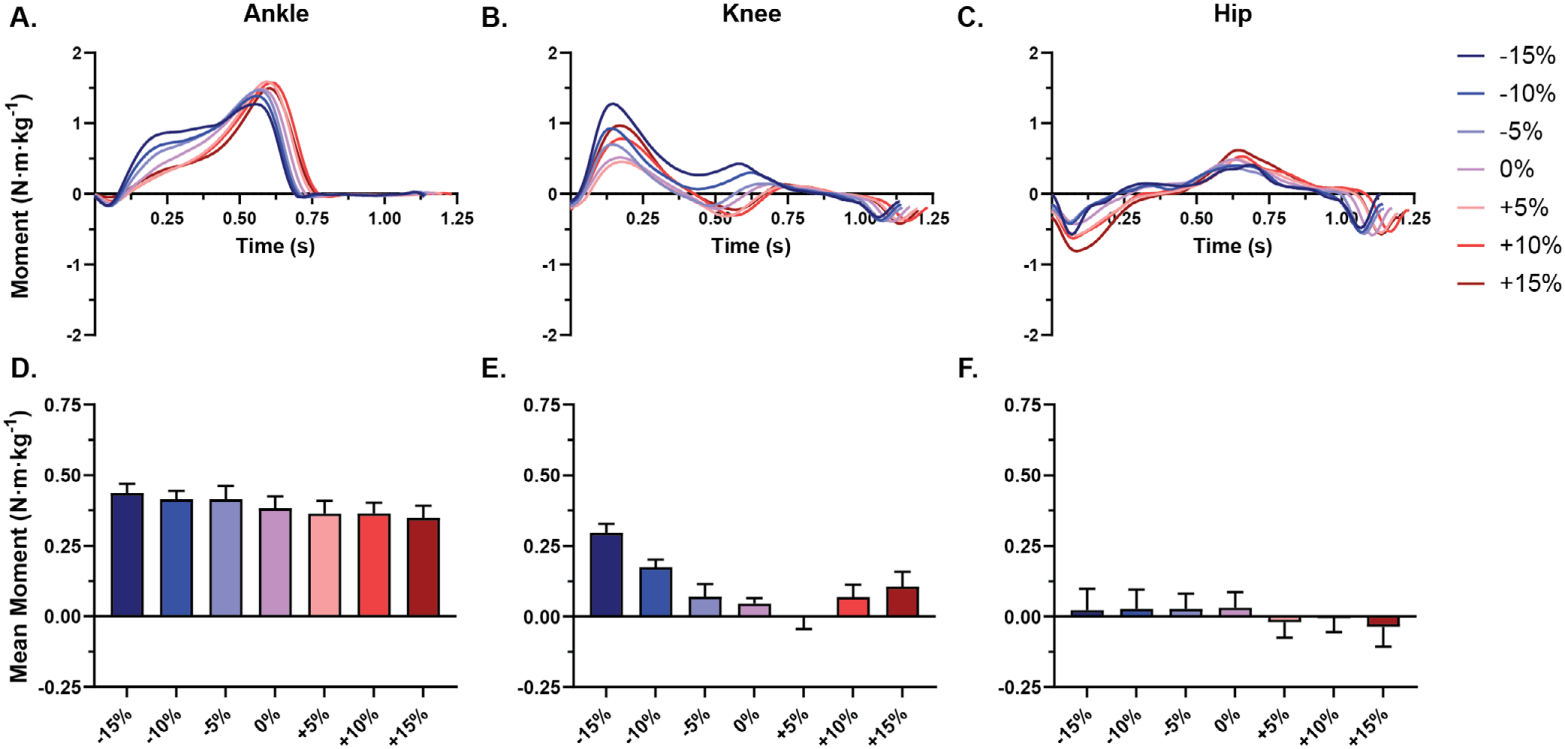
Comparison of right‐leg (A) ankle, (B) knee, and (C) hip joint moments (N·m·kg^−1^) across the gait cycle (real‐time, right‐foot ground contact through to the end of the right‐foot swing phase), and (D) ankle, (E) knee, and (F) hip mean ± SD moment across all prescribed walking conditions (−15%, −10%, −5%, 0%, +5%, +10%, +15%). Data averaged across 12 participants.

Net joint work increased as slope became more positive (Figure 3). Strong correlations were observed between net joint work and muscle work costs at the ankle (*β* = 2.02, 95% CI [1.83, 2.22], adjusted *R* ^2^ = 0.88), knee (*β* = 2.17, 95% CI [1.92, 2.42], adjusted *R* ^2^ = 0.83), and hip (*β* = 3.19, 95% CI [2.74, 3.63], adjusted *R* ^2^ = 0.77). Furthermore, including net joint work from all three joints as predictors in the regression model explained nearly all variance in muscle work costs (adjusted *R* ^2^ = 0.95) (Figure S6B). Notably, positive joint work—particularly at the knee—increased at steeper uphill slopes and plateaued at level and downhill slopes, reflecting the pattern of total metabolic rate (Figure 1). Including positive joint work from all three joints in a regression model of experimental metabolic rate yielded a very strong correlation (adjusted *R* ^2^ = 0.92) (Figure S6C).

**FIGURE 3:**
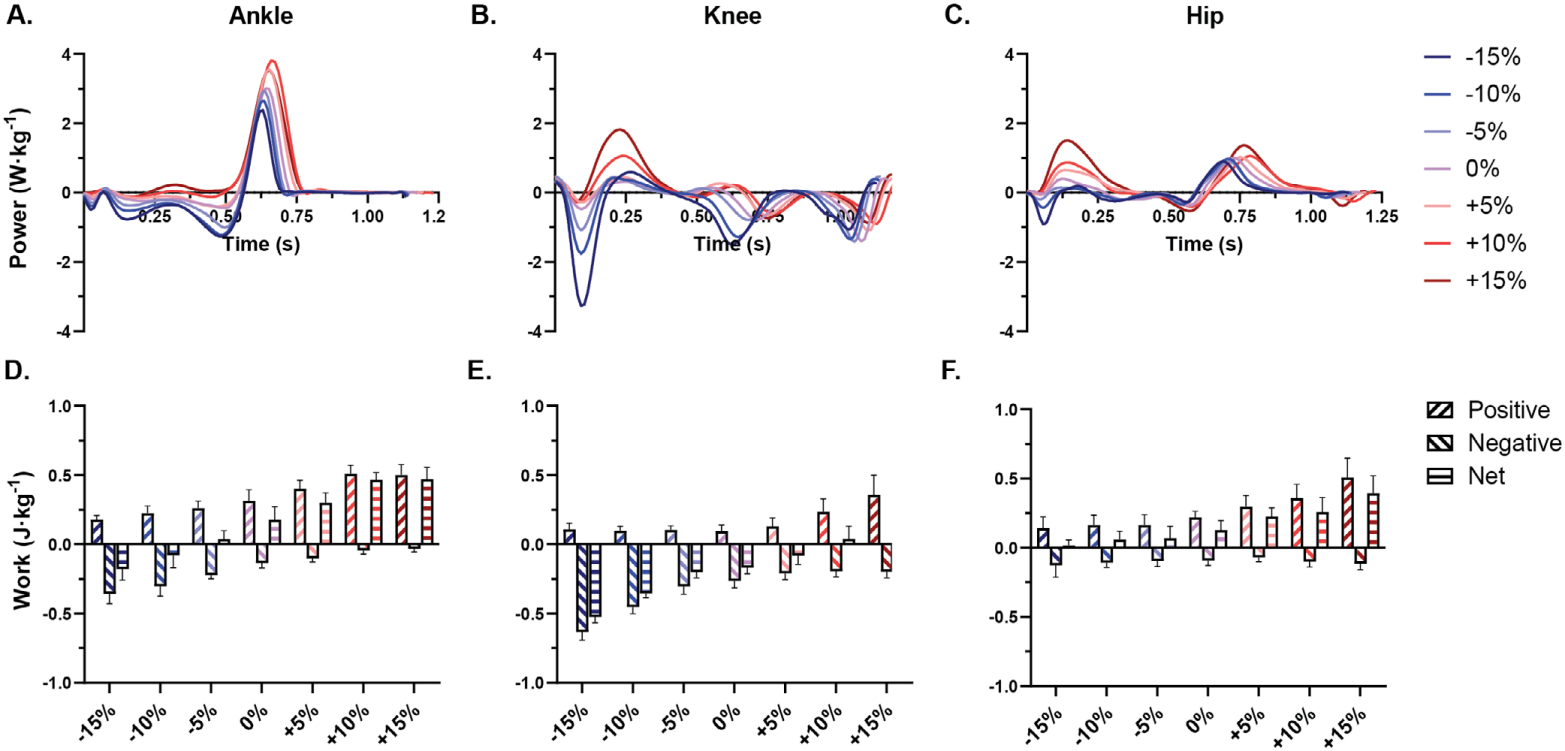
Comparison of right‐leg (A) ankle, (B) knee, and (C) hip joint powers (W·kg^−1^) across the gait cycle (real‐time, from right‐foot ground contact through to the end of the right‐foot swing phase), and (D) ankle, (E) knee, and (F) hip mean ± SD positive, negative, and net joint work (J·kg^−1^) across all prescribed walking conditions (−15%, −10%, −5%, 0%, +5%, +10%, +15%). Data averaged across 12 participants.

## 4 Discussion

We used a musculoskeletal modeling approach to simulate muscle mechanics and energetics during sloped walking between ±15% slope. Although predictions of total metabolic rates differed substantially in absolute terms from experimental data, considering only the energy expenditure of the major work‐producing muscles in the model yielded very strong correlations between simulated and experimental metabolic rates. As hypothesized, muscle force‐related costs (*ḣ*_A_ + *ḣ*_M_) increased with steeper uphill and downhill slopes, while muscle work‐related costs (*ẇ*_CE_ + *ḣ*_SL_) increased with more positive slopes. In our simulations, force costs dominated changes in metabolic rate during downhill walking, with work costs contributing more during uphill walking.

Simulated muscle force costs (Figure 1B) were predicted reasonably well by changes in mean joint moment (Figures 2D–F and S6A). Consistent with our hypothesis that knee mechanics would be particularly indicative of variance in costs, we observed a similar U‐shaped relationship between force costs and slope as for knee mean joint moment, increasing at steeper uphill and downhill slopes. In contrast, ankle and hip mean joint moments changed little with slope. This was reflected in the muscle contributions to total force costs across conditions (Figure 4), where VL and RF showed the largest changes among the muscles that were evaluated. Notably, this occurred despite clear changes in ankle and hip muscle activation across slopes (Figure S1), consistent with the findings from Lay et al. [20] and Franz and Kram [21]. However, changes in activation, mean moment, and force costs occurred on a much smaller scale at the ankle and hip compared to the knee.

**FIGURE 4:**
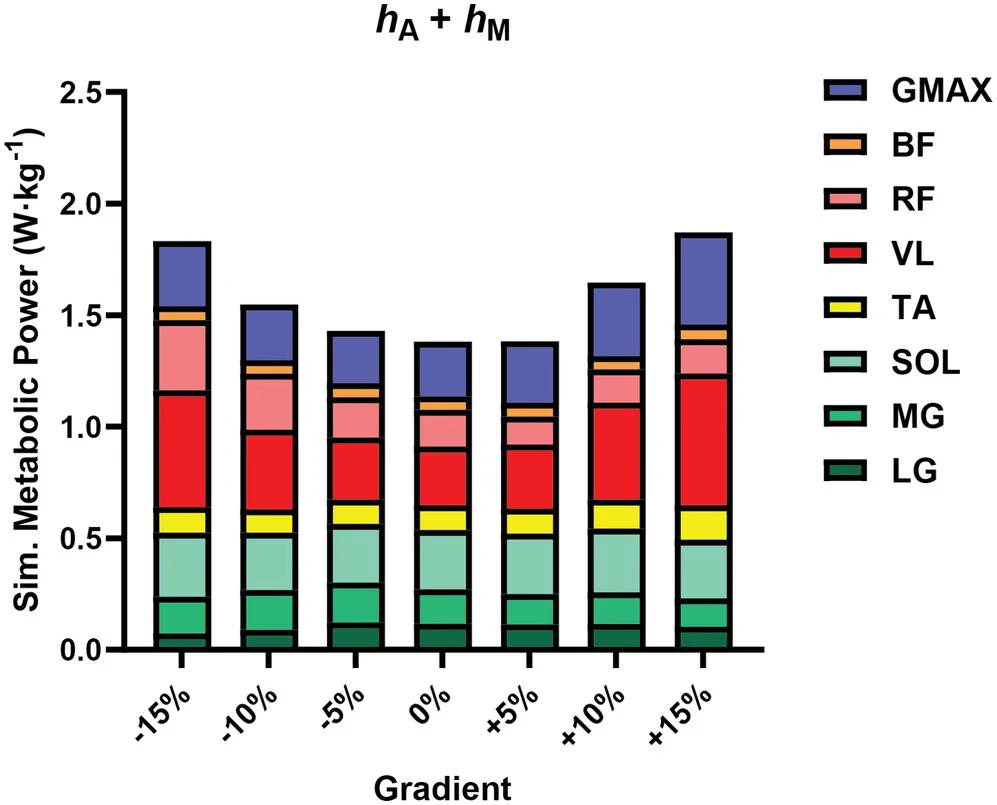
Comparison between the contribution of lateral gastrocnemius (LG), medial gastrocnemius (MG), soleus (SOL), tibialis anterior (TA), vastus lateralis (VL), rectus femoris (RF), biceps femoris (BF), and gluteus maximus (GMAX), summed bilaterally, to total net force‐related costs (activation heat (*ḣ*_A_) + maintenance heat (*ḣ*_M_)) (W·kg^−1^) across all prescribed walking conditions (−15%, −10%, −5%, 0%, +5%, +10%, +15%) from the Umberger cost model. Data averaged across 12 participants.

We also observed that simulated muscle work costs increased with more positive slopes (Figure 1C), and were predicted extremely well by net joint work per stride at the ankle, knee, and hip (Figures 3D–F and S6B). Based on net joint work, we infer substantial contributions from all three joints to changes in total work costs. As slope became more positive from −15% to +15%, LG, SOL, and VL fascicles went from lengthening more to shortening more during stance (Figure S2). Furthermore, total metabolic rate correlated very strongly with changes in positive joint work (Figure S6C). This aligns with collisional models that base cost calculations on the positive work required to offset energy dissipation during stance, often neglecting isometric and eccentric costs [6, 49]. We also noted a plateau in positive joint work as slope became more negative from 0% to −15% slope (Figure 3D–F), likely reflecting increased force demands at steeper declines. Nonetheless, there were slight differences between positive joint work and total metabolic rate, such as the location of the energetic minimum and the uptick in total metabolic rate at steeper downhill slopes.

Although joint work explained nearly all variance in muscle work costs, joint moments were only moderately correlated with force costs. This likely reflects that costs of generating force depend not just on the magnitude of force but also on muscle operating length, per the force‐length relationship [10, 50, 51]. For example, in Figure 5, VL activation and knee moments are higher at −15% than at 0% slope, yet VL fascicle lengths during the main period of force production were closer to optimal (i.e., nearer to 1.0) at −15% slope. We used mean joint moments as proxies for average force requirements per unit time [52, 53]. Because participants maintained similar gait cycle durations and duty factors across conditions, other metrics of force and time used to model the cost of generating force (e.g., force rate [35, 52, 54, 55], time integral of force [36, 56]) are unlikely to have improved our joint‐level predictions of muscle force costs. Current force‐based cost models may thus struggle to explain all variance in force costs under varying muscle length conditions.

**FIGURE 5:**
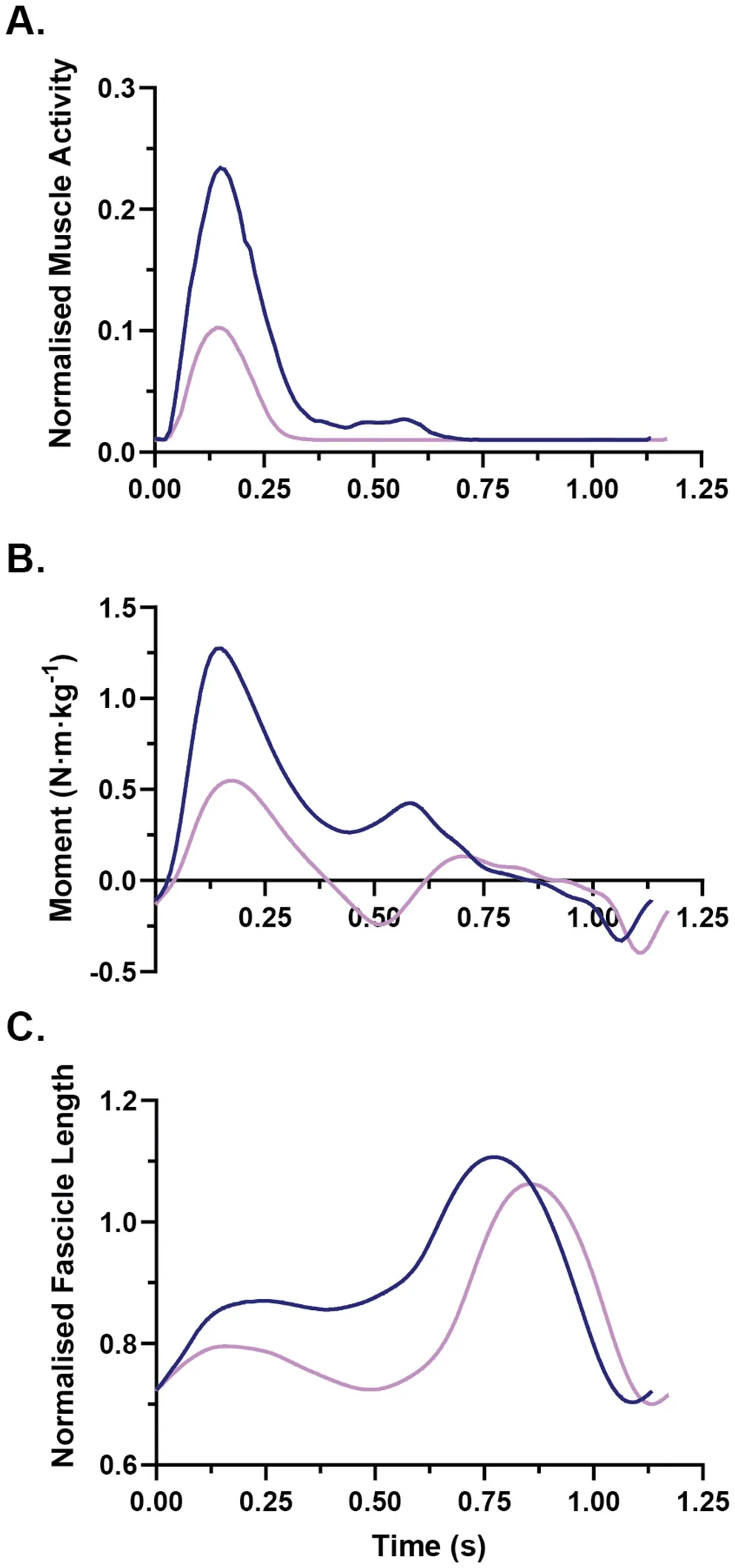
Comparison of time‐series (A) simulated normalized muscle activity of vastus lateralis (VL), (B) knee joint moment (N·m·kg^−1^), and (C) simulated normalized fascicle length of VL between walking at −15% (blue) and 0% (purple) grade. Data are from the right leg, with both experimental and simulated data averaged across 12 participants.

Our findings have important implications for biomechanical models of locomotor energetics. During sloped walking, mechanical work‐ and collision‐based models (e.g., [2, 3, 5]) can capture system‐level relationships between work and metabolic cost. However, incorporating a force‐related cost term may provide a more mechanistic link between muscle mechanical demands and both the costs of downhill walking and the nonlinearity of the slope‐cost curve. Conversely, force‐based frameworks alone, which relate metabolic cost to the force required to support body weight and the time available to produce it [35, 36, 52, 54, 55, 56], would likely underestimate the additional cost of uphill locomotion. Combined approaches that include both force‐ and work‐related terms [38, 57] are therefore more consistent with our simulations, which suggest that force‐ and work‐related costs contribute differently across slopes. Hoogkamer et al. [57] achieved better predictions of uphill running energy expenditure by including both force‐ and work‐based costs. Our results extend these frameworks by indicating which muscles and joints may underlie each cost component, with knee mechanics emerging as a potentially useful indicator of energy costs during sloped walking. However, our participants walked at a single speed with similar gait parameters (Table 1); whether varying parameters such as stride frequency, step width, and foot clearance alters joint‐level contributions to activation and work demands, and the shape of the slope‐cost curve, warrants further investigation [21, 58, 59, 60].

This is the first study to evaluate a complete three‐dimensional lower‐limb musculoskeletal model for simulating muscle mechanics and energetics during uphill and downhill walking. We found good agreement between salient features of experimental and simulated muscle activations (Figure S1) and fascicle dynamics (Figure S2) for key muscles (LG, MG, SOL, TA, VL, RF, BF, and GMAX) across slopes. However, absolute differences in metabolic power were large, with overestimation in all but the steepest uphill conditions when considering all muscles in the model (Figure S3). This contrasts with Koelewijn et al. [29], who reported smaller absolute differences for most cost models and underestimation of energy cost between ±8% slope. Methodological differences that may contribute include their use of a two‐dimensional sagittal‐plane model, a custom muscle model, and eight muscles per leg whose parameters (e.g., maximum isometric force) may have been scaled to account for muscles not modeled. When we limited analyses to the muscles we evaluated with EMG and ultrasound, simulated and experimental slope‐cost curves were much more strongly correlated (Figure 1A; Figure S5B), suggesting that the mechanics of other muscles may have been simulated less accurately—possibly due to the objective function, measurement error, or broader limitations of current modeling approaches. Restricting analyses to a subset of the main power‐producing muscles likely reduced some sources of error, but it also may have omitted important costs, underestimating metabolic power by ~30%–60% across grades. While this subset is expected to have captured most sagittal‐plane costs, it, along with locking the subtalar joint (see Supporting Information), probably excluded substantial frontal‐plane costs. Pickle et al. [61] found no change in mediolateral centre of mass (CoM) accelerations or gluteus medius EMG with slope. Moreover, data from Fujimoto et al. [62] suggest only a modest increase in peroneus longus EMG during downhill walking. However, because relevant fascicle data are currently unavailable for such muscles, it is difficult to infer whether frontal‐plane costs remain consistent across grades and how they might influence the shape of the slope‐cost relationship. Ultimately, frontal‐plane mechanics during graded locomotion remain understudied, and readers should interpret our findings with these simplifications in mind.

There are additional limitations related to the analysis. The sample size of this study is modest (*n* = 12), though comparable to other studies combining musculoskeletal simulation with experimental indirect calorimetry, ultrasound and/or EMG measurements [29, 32, 33, 34]. Furthermore, each participant contributed multiple slope conditions (68 trials in total; 4–7 per participant), and including participant identity in the regression models meant associations were estimated from within‐participant changes across slopes. Interindividual differences accounted for negligible variance in experimental metabolic power and simulated muscle work costs (participant‐only models: adjusted *R* ^2^ < 0) and a modest proportion of variance in simulated muscle force costs (adjusted *R* ^2^ = 0.14). Accordingly, confidence intervals for the principal relationships were relatively narrow (e.g., simulated vs. experimental metabolic power: *β* = 4.11, 95% CI [3.87, 4.36]). Nonetheless, larger samples would allow interindividual differences in these relationships to be examined, such as whether the sensitivity of muscle costs to joint mechanics varies with gait characteristics. Finally, the relationships reported here are correlational; experiments that manipulate factors directly (e.g., by modifying work or force requirements) would be needed to better establish the causal link.

The muscle and metabolic cost models used here also have limitations. Using a Hill‐type muscle model enabled efficient simulation of gross MTU behavior [63] but sacrificed finer structural and functional details affecting energy expenditure, such as muscle‐specific histology [64], shape changes [65], nonuniform tendon dynamics [66], and history dependence [67]. The precise cost of eccentric work also remains unresolved and a major point of contention among metabolic cost models (for a review, see Miller et al. [27]). Although the cost model we used includes terms for eccentric work, different treatments of eccentric work substantially affect predictions of locomotor energetics [27, 68]. Moreover, these models do not account for energy costs of ATP regeneration via glycolysis or oxidative recovery, which releases heat over longer timescales than the contractions themselves [69]. Lastly, motor unit recruitment dynamics are represented in a simplified form, neglecting the fact that these dynamics and their associated costs—potentially linked to recovery processes and Ca^2+^ transport [70]—vary with muscles' mechanical state [71].

Our modeling approach allowed us to estimate muscle force‐ and work‐related costs to help interpret different features of the slope‐cost curve of walking: simulated force costs were associated with downhill walking and non‐linearity in the curve, and work costs with the greater cost of uphill walking. While joint work explained variance in work costs, joint moments only moderately correlated with force costs, likely due to variations in muscle operating lengths. Compared to traditional mechanical work‐based cost models, including a force‐based term may yield more explanatory models that better map to muscle mechanical demands. Knee mechanics appear to be particularly good indicators of total energy costs during sloped walking, and future work should examine how changes in gait parameters affect joint‐ and muscle‐level demands and reshape the slope‐cost curve.

## 5 Perspective

Future research should experimentally manipulate force and work demands to test whether the slope‐cost relationship changes in the way our correlational findings suggest. For example, if these relationships are causal, reducing force demands would be expected to lower costs at steep slopes, especially declines, whereas reducing positive work demands should mainly lower costs on inclines. At a given slope, these demands could be shifted by constraining step frequency or length, adding load or body‐weight support, using exoskeletons that redistribute work between joints, or providing EMG‐based biofeedback to alter muscle activation. Such manipulations may also reveal whether knee mechanics remain a useful indicator of energy cost across a wider range of sloped walking conditions. Additionally, measuring activation and fascicle behavior in frontal‐plane muscles, and using these data to inform models, may help determine whether frontal‐plane costs contribute to the slope‐cost relationship and improve absolute predictions of metabolic cost. Together, these approaches should provide a more mechanistic basis for predicting energy expenditure across terrains, and inform strategies (e.g., assistive devices or gait retraining) to reduce the energetic demands of locomotion.

## Author Contributions

**Luke N. Jessup:** conceptualization, formal analysis, investigation, methodology, project administration, visualization, writing – original draft, writing – review and editing; **Luke A. Kelly:** conceptualization, methodology, supervision, writing – review and editing; **Andrew G. Cresswell:** methodology, supervision, writing – review and editing; **Glen A. Lichtwark:** conceptualization, funding acquisition, methodology, project administration, resources, supervision, writing – review and editing.

## Funding

L.N.J. was supported by a University of Queensland Graduate Student Scholarship during the majority of this work and is currently supported by the U.S. National Science Foundation (NSF‐DBI‐2319710). G.A.L. and research costs were supported by the Australian Research Council (FT190100129). L.A.K. was supported by the Australian Research Council (DE200100585).

## Conflicts of Interest

Andrew G. Cresswell is a Senior Section Editor of Scandinavian Journal of Medicine & Science in Sports. The other authors declare no conflicts of interest.

## Supporting information


**Data S1:** Supporting Information.

**Data S2:** Supporting Information.

**Figure S1:** Comparison between normalized experimental EMG and normalized simulated muscle activity across the gait cycle (time‐normalized from right‐foot ground contact through to the end of the right‐foot swing phase) and across all prescribed walking conditions (−15%, −10%, −5%, 0%, +5%, +10%, +15%; dark blue to dark red, respectively) for right‐leg (A) lateral gastrocnemius (LG), (B) medial gastrocnemius (MG), (C) soleus (SOL), (D) tibialis anterior (TA), (E) vastus lateralis (VL), (F) rectus femoris (RF), (G) biceps femoris (BF) and (H) gluteus maximus (GMAX). Data averaged across seven participants.
**Figure S2:** Comparison between normalized experimental and simulated fascicle length changes across the gait cycle (time‐normalized from right‐foot ground contact through to the end of the right‐foot swing phase) and across all prescribed walking conditions (−15%, −10%, −5%, 0%, +5%, +10%, +15%) for right‐leg (A) lateral gastrocnemius (LG), (B) soleus (SOL) and vastus lateralis (VL) (C). Data averaged across seven participants.
**Figure S3:** Comparison between mean ± SD experimental net metabolic power (W·kg^−1^) (*Exp*.; circles; solid lines; primary *Y*‐axis), simulated net metabolic power summed from all muscles in the musculoskeletal model (*Sim. All*; squares; solid lines; primary *Y*‐axis) and simulated net metabolic power summed bilaterally from only lateral gastrocnemius, medial gastrocnemius, soleus, tibialis anterior, vastus lateralis, rectus femoris, biceps femoris and gluteus maximus in the musculoskeletal model (*Sim. Select*; diamonds; dashed lines; secondary *Y*‐axis) across each of the prescribed walking conditions (−15%, −10%, −5%, 0%, +5%, + 10%, +15%). *Exp*. and *Sim. All* data are averaged across seven participants, while *Sim. Select* data are averaged across 12 participants.
**Figure S4:** Mean ± SD contribution (%) of each muscle in the model to changes in simulated net metabolic power, summed bilaterally, across the graded walking conditions. Data averaged across seven participants.
**Figure S5:** Linear models between experimental metabolic power (W·kg^−1^) and (A) simulated net metabolic power based on all muscles in the model and (B) simulated net metabolic power summed bilaterally based on only lateral gastrocnemius, medial gastrocnemius, soleus, tibialis anterior, vastus lateralis, rectus femoris, biceps femoris and gluteus maximus in the model. Participant was used as a categorical predictor in these linear models. Data are from 12 participants.
**Figure S6:** Linear models between (A) muscle force costs (W·kg−1) and combined (ankle, knee and hip) mean joint moment (N·m·kg−1), (B) muscle work costs (W·kg−1) and combined net joint work (J·kg−1) and (C) total cost (W·kg−1) and combined positive joint work (J·kg−1). Participant was used as a categorical predictor in these linear models. Simulated data were summed bilaterally from lateral gastrocnemius, medial gastrocnemius, soleus, tibialis anterior, vastus lateralis, rectus femoris, biceps femoris and gluteus maximus. Data are from 12 participants.

## Data Availability

The data that support the findings of this study are available in the Supporting Information of this article. These include, for each participant and condition, the mass‐normalized experimental and simulated metabolic power and experimental joint moment and work data used in the regression analyses. They also include the averaged experimental and simulated normalized muscle activation and fascicle length data used for model evaluation.
