# Supplementary material for: Dissecting the Metabolic Costs of Uphill and Downhill Walking
Source: Scand J Med Sci Sports. 2026 Oct 26;36(11):e70389. doi: 10.1111/sms.70389 (PMC13702041; doi:10.1111/sms.70389)
Supplement: Supplementary file 1 [file SMS-36-e70389-s001.docx]

**Supplementary materials**

Here we detail the model refinement and evaluation procedures used in the study, “Dissecting the Metabolic Costs of Uphill and Downhill Walking.”

The open-source model published by Lai et al. [1] was used at the base model for our analyses because of its design to reduce passive forces when simulating movements that involve substantial knee and hip flexion (e.g., steep uphill walking). To verify that the experimental motion capture data were being correctly applied to the model, joint kinematics and kinetics were compared qualitatively, in shape, timing, and magnitude, to sloped walking data reported in the literature. We also ensured that the reserve moments (the difference between net joint moments and muscle-generated net joint moments) were no greater than 5% of the peak net joint moments, per recommendations [2].

We first used an automated approach to optimise the muscle properties of each participant’s model [3]; scaled the maximum isometric force of all muscles based on the mass of the participant relative to the mass of the generic model; adjusted each model’s lateral gastrocnemius, soleus, and vastus lateralis optimal fascicle lengths to the respective mean fascicle lengths to which we normalised our experimental data; adjusted the tendon slack lengths to account for these changes in optimal fascicle length (to maintain a similar joint angle at which muscle fascicle length first starts to increase when the joint is passively rotated); and converted the muscle model type to *DeGrooteFregly2016Muscle* [4]. We then went through an iterative, systematic process to determine whether any model properties could be manually adjusted to reduce absolute differences and improve the qualitative similarity of salient features between our experimental data and simulated muscle activations (Fig. S1), fascicle dynamics (Fig. S2), and whole-body metabolic powers (Fig. S3). This “evaluation” of the modelling was performed on seven of our thirteen participants who varied in mass and sex (61-92 kg; 3 female). The purpose of this “evaluation” was to identify systematic model deficiencies rather than to characterise individual responses, and because simulated outputs were highly consistent across these participants, including additional participants was unlikely to alter the outcome of the evaluation. This is consistent with our previous evaluation of a similar modelling approach, in which simulated muscle activations and fascicle dynamics agreed well with experimental data across diverse hopping tasks [5].

Due to the computational demands of Moco, we simulated one gait cycle per trial, per participant. The cycle was selected by overlaying each individual cycle's left and right joint moments on the average across the three experimental cycles and visually identifying the cycle most similar in magnitude and shape. This was intended to ensure the simulated cycle was representative of the participant's steady-state gait in that condition. Although this approach does not capture stride-to-stride variability, such within-condition variability in EMG, fascicle behaviour, and joint mechanics appeared small relative to the changes across slopes, as expected given that data were analysed from periods of steady-state walking. Simulated muscle activations and fascicle lengths were collated for the same muscles measured experimentally for the evaluation. For each of the seven evaluated participants, experimental EMGs were normalized to the maximum experimental EMG observed for each muscle across all trials performed by that participant, and simulated muscle activations were normalized to the maximum simulated activation for the corresponding muscle across the same trials. We assumed that the simulated muscle activations were analogous to the filtered excitation signals measured directly from the muscle (enveloped EMG). Both the experimental and simulated fascicle lengths were normalised to the respective muscle’s mean experimental fascicle length across all trials that the participant performed.

Whole-body metabolic power was estimated from the simulations performed on those seven participants using the cost model developed by Umberger et al. [6] with some modifications [7,8]. The Umberger model was implemented via OpenSim’s probe reporter tool using the *Umberger2010MuscleMetabolicsProbe*. To compute gross whole-body metabolic power, the controls and states from Moco were fed to the cost model, we then summed the computed rate of energy expenditure across all muscles, plus the outputted whole-body mass-specific basal rate, and then integrated the resulting whole-body metabolic rate over the gait cycle and divided by the duration of the gait cycle.

During the process of model refinement within our evaluation to achieve suitable muscle activations and fascicle length changes, we found it necessary to remove the gastrocnemii femoral and tibial wrapping surfaces from the model because they led to overactivation and discontinuities in fascicle length changes of the gastrocnemii. We also locked the subtalar and metatarsophalangeal joints to prevent unrealistic motion that otherwise caused overactivation of the muscles crossing these joints. This is a known issue, commonly addressed in this way [5,9-11]: standard whole-body motion capture lacks the resolution to accurately track these small degrees of freedom, producing large spurious accelerations that require joint moments beyond the capacity of the muscles that cross them. Further, we added reserve actuators to each model degree-of-freedom with a maximum force of 10.0 N or Nm to combat any remaining dynamic inconsistency in the modelling.

1. A. Lai, A. S. Arnold, and J. M. Wakeling, “Why Are Antagonist Muscles Co-Activated in My Simulation? A Musculoskeletal Model for Analysing Human Locomotor Tasks,” *Annals of Biomedical Engineering* 45, no. 12 (2017): 2762–2774, https://doi.org/10.1007/s10439-017-1920-7.
2. J. L. Hicks, T. K. Uchida, A. Seth, A. Rajagopal, and S. L. Delp, “Is My Model Good Enough? Best Practices for Verification and Validation of Musculoskeletal Models and Simulations of Movement,” *Journal of Biomechanical Engineering* 137, no. 2 (2015): 020905, https://doi.org/10.1115/1.4029304.
3. L. Modenese, E. Ceseracciu, M. Reggiani, and D. G. Lloyd, “Estimation of Musculotendon Parameters for Scaled and Subject Specific Musculoskeletal Models Using an Optimization Technique,” *Journal of Biomechanics* 49, no. 2 (2016): 141–148, https://doi.org/10.1016/j.jbiomech.2015.11.006.
4. F. De Groote, A. L. Kinney, A. V. Rao, and B. J. Fregly, “Evaluation of Direct Collocation Optimal Control Problem Formulations for Solving the Muscle Redundancy Problem,” *Annals of Biomedical Engineering* 44, no. 10 (2016): 2922–2936, https://doi.org/10.1007/s10439-016-1591-9.
5. L. N. Jessup, L. A. Kelly, A. G. Cresswell, and G. A. Lichtwark, “Validation of a Musculoskeletal Model for Simulating Muscle Mechanics and Energetics During Diverse Human Hopping Tasks,” *Royal Society Open Science* 10, no. 10 (2023): 230393, https://doi.org/10.1098/rsos.230393.
6. B. R. Umberger, K. G. Gerritsen, and P. E. Martin, “A Model of Human Muscle Energy Expenditure,” *Computer Methods in Biomechanics and Biomedical Engineering* 6, no. 2 (2003): 99–111, https://doi.org/10.1080/1025584031000091678.
7. B. R. Umberger, “Stance and Swing Phase Costs in Human Walking,” *Journal of the Royal Society Interface* 7, no. 50 (2010): 1329–1340, https://doi.org/10.1098/rsif.2010.0084.
8. T. K. Uchida, J. L. Hicks, C. L. Dembia, and S. L. Delp, “Stretching Your Energetic Budget: How Tendon Compliance Affects the Metabolic Cost of Running,” *PLoS One* 11, no. 3 (2016): e0150378, https://doi.org/10.1371/journal.pone.0150378.
9. G. B. De Carvalho, V. D. Chandran, A. M. Spungen, N. Y. Harel, W. A. Bauman, and S. Pal, “Hip, Knee, and Ankle Joint Forces During Exoskeletal-Assisted Walking: Comparison of Approaches to Simulate Human-Robot Interactions,” *PLoS One* 20, no. 8 (2025): e0322247, https://doi.org/10.1371/journal.pone.0322247.
10. A. Rajagopal, C. L. Dembia, M. S. DeMers, D. D. Delp, J. L. Hicks, and S. L. Delp, “Full-Body Musculoskeletal Model for Muscle-Driven Simulation of Human Gait,” *IEEE Transactions on Biomedical Engineering* 63, no. 10 (2016): 2068–2079, https://doi.org/10.1109/TBME.2016.2586891.
11. A. M. Willson, A. J. Anderson, C. A. Richburg, et al., “Full Body Musculoskeletal Model for Simulations of Gait in Persons with Transtibial Amputation,” *Computer Methods in Biomechanics and Biomedical Engineering* 26, no. 4 (2023): 412–423, https://doi.org/10.1080/10255842.2022.2065630.

**Figure S1.** Comparison between normalised experimental EMG and normalised simulated muscle activity across the gait cycle (time-normalised from right-foot ground contact through to the end of the right-foot swing phase) and across all prescribed walking conditions (-15%, -10%, -5%, 0%, +5%, +10%, +15%; dark blue to dark red, respectively) for right-leg (**A**) lateral gastrocnemius (LG), (**B**) medial gastrocnemius (MG), (**C**) soleus (SOL), (**D**) tibialis anterior (TA), (**E**) vastus lateralis (VL), (**F**) rectus femoris (RF), (**G**) biceps femoris (BF) and (**H**) gluteus maximus (GMAX). Data averaged across seven participants.

**Figure S2.** Comparison between normalised experimental and simulated fascicle length changes across the gait cycle (time-normalised from right-foot ground contact through to the end of the right-foot swing phase) and across all prescribed walking conditions (-15%, -10%, -5%, 0%, +5%, +10%, +15%) for right-leg (**A**) lateral gastrocnemius (LG), (**B**) soleus (SOL) and vastus lateralis (VL) (**C**). Data averaged across seven participants.

**Figure S3.** Comparison between mean ± SD experimental net metabolic power (W·kg^-1^) (*Exp.*; circles; solid lines; primary Y-axis), simulated net metabolic power summed from all muscles in the musculoskeletal model (*Sim. All*; squares; solid lines; primary Y-axis) and simulated net metabolic power summed bilaterally from only lateral gastrocnemius, medial gastrocnemius, soleus, tibialis anterior, vastus lateralis, rectus femoris, biceps femoris and gluteus maximus in the musculoskeletal model (*Sim. Select*; diamonds; dashed lines; secondary Y-axis) across each of the prescribed walking conditions (-15%, -10%, -5%, 0%, +5%, +10%, +15%). *Exp.* and *Sim. All* data are averaged across seven participants, while *Sim. Select* data are averaged across twelve participants.

**Figure S4.** Mean ± SD contribution (%) of each muscle in the model to changes in simulated net metabolic power, summed bilaterally, across the graded walking conditions. Data averaged across seven participants.

**Figure S5.** Linear models between experimental metabolic power (W·kg^-1^) and (**A**) simulated net metabolic power based on all muscles in the model and (**B**) simulated net metabolic power summed bilaterally based on only lateral gastrocnemius, medial gastrocnemius, soleus, tibialis anterior, vastus lateralis, rectus femoris, biceps femoris and gluteus maximus in the model. Participant was used as a categorical predictor in these linear models. Data are from twelve participants.

**Figure S6.** Linear models between (**A**) muscle force costs (W·kg^-1^) and combined (ankle, knee and hip) mean joint moment (N·m·kg^-1^), (**B**) muscle work costs (W·kg^-1^) and combined net joint work (J·kg^-1^) and (**C**) total cost (W·kg^-1^) and combined positive joint work (J·kg^-1^). Participant was used as a categorical predictor in these linear models. Simulated data were summed bilaterally from lateral gastrocnemius, medial gastrocnemius, soleus, tibialis anterior, vastus lateralis, rectus femoris, biceps femoris and gluteus maximus. Data are from twelve participants.
